# Cytoxicity and Apoptotic Mechanism of Ruthenium(II) Amino Acid Complexes in Sarcoma-180 Tumor Cells

**DOI:** 10.1371/journal.pone.0105865

**Published:** 2014-10-17

**Authors:** Aliny Pereira Lima, Flávia Castro Pereira, Marcio Aurelio Pinheiro Almeida, Francyelli Mariana Santos Mello, Wanessa Carvalho Pires, Thallita Monteiro Pinto, Flávia Karina Delella, Sérgio Luis Felisbino, Virtudes Moreno, Alzir Azevedo Batista, Elisângela de Paula Silveira-Lacerda

**Affiliations:** 1 Laboratory of Molecular Genetics and Cytogenetics, Institute of Biological Sciences, University Federal of Goiás-UFG, Goiânia, Goiás, Brazil; 2 Coordination of Science and Technology, University Federal of Maranhão, São Luís, Maranhão, Brazil; 3 Department of Chemistry, University Federal of São Carlos, São Carlos, São Paulo, Brazil; 4 Department of Morphology, Institute of Biosciences – University Estadual Paulista (UNESP), Botucatu, São Paulo, Brazil; 5 Department of Inorganic Chemistry, Universitat de Barcelona, Barcelona, Spain; Wayne State University, United States of America

## Abstract

Over the past several decades, much attention has been focused on ruthenium complexes in antitumor therapy. Ruthenium is a transition metal that possesses several advantages for rational antitumor drug design and biological applications. In the present study, five ruthenium complexes containing amino acids were studied *in vitro* to determine their biological activity against sarcoma-180 tumor cells. The cytotoxicity of the complexes was evaluated by an MTT assay, and their mechanism of action was investigated. The results demonstrated that the five complexes inhibited the growth of the S180 tumor cell line, with IC_50_ values ranging from 22.53 µM to 50.18 µM, and showed low cytotoxicity against normal L929 fibroblast cells. Flow cytometric analysis revealed that the [Ru(gly)(bipy)(dppb)]PF_6_ complex (**2**) inhibited the growth of the tumor cells by inducing apoptosis, as evidenced by an increased number of Annexin V-positive cells and G0/G1 phase cell cycle arrest. Further investigation showed that complex 2 caused a loss of mitochondrial membrane potential; activated caspases 3, caspase-8, and caspase-9 and caused a change in the mRNA expression levels of caspase 3, caspase-9 as well as the bax genes. The levels of the pro-apoptotic Bcl-2 family protein Bak were increased. Thus, we demonstrated that ruthenium amino acid complexes are promising drugs against S180 tumor cells, and we recommend further investigations of their role as chemotherapeutic agents for sarcomas.

## Introduction

Sarcomas constitute a heterogeneous group of rare solid tumors of mesenchymal cell origin with distinct clinical and pathological features, and they are usually divided into 2 broad categories: sarcomas of soft tissues (including fat, muscle, nerve, nerve sheath, blood vessels, and other connective tissues) and sarcomas of the bone. Collectively, sarcomas account for approximately 1% of all adult and 15% of all pediatric malignancies [1].

Anthracyclines and ifosfamide have been established as the most active drugs for the treatment of patients with advanced soft tissue sarcomas of most histologic subtypes, with the exception of gastrointestinal stromal tumors. However, after failure of these drugs, patients with advanced soft tissue sarcomas have few treatment options, and the limitation of current therapeutic options for these tumors has prompted the development and evaluation of a very large number of new chemotherapeutic and biological agents for their treatment [2]. Thus, the development of new drugs to treat cancer in general and sarcoma specifically is required. Ruthenium complexes represent a new family of promising metal-based anticancer drugs that offer the potential for reduced toxicity compared to the antitumor platinum(II) complexes currently used in the clinical setting. These complexes have a novel mechanism of action, are less likely to exhibit cross-resistance, and have a different spectrum of activity compared to the platinum drugs [3]–[6].

The mechanism by which ruthenium complexes exert anticancer effects has been widely investigated. Several mechanisms have been suggested to underlie the anticancer activities of ruthenium complexes, including the inhibition of metastasis [7], [8] interaction with DNA [9], interaction with proteins [10], production of reactive oxygen species [11], inhibition of topoisomerase activity [12] and induction of apoptosis [13]. In addition, the mechanisms by which ruthenium complexes exert anticancer effects have been shown to largely depend on the nature of the complexes, the ligands involved, and the presence of uncoordinated sites in the coordination sphere of the metal center.

In the search for selective and effective complexes that can be targeted against cancer, our research group synthesized several ruthenium(II) complexes with different ligands [14]–[16], e.g., ruthenium complexes coordinated with amino acids, which have demonstrated cytotoxic activity against the model of murine breast cancer, MDA-MB-231 cells. Based on these observations, the aim of the present study was to examine the cytotoxic effects of the five ruthenium complexes containing amino acid ligands against the S180 murine sarcoma cell line *in vitro* and to elucidate the molecular mechanism by which ruthenium/amino acid complexes cause cancer cell death and induce cell cycle perturbations. The complexes evaluated in this study were [Ru(AA)(bipy)(dppb)]PF_6_, where AA  =  methionine (complex 1), glycine (complex 2), leucine (complex 3), aspartic acid (complex 4), or alanine (complex 5) bipy  =  2,2′-bipyridine; and dppb  =  [1,4-bis(diphenylphosphine)butane].

In this study, the [Ru(gly)(bipy)(dppb)]PF_6_, [complex 2], was the most promising species tested, showing the highest activity against the S180 tumor cells and low cytotoxicity against L929 normal cells. Thus, this complex was selected for further investigation to determine its mechanism of action against sarcoma-180 tumor cells.

## Materials and Methods

### Chemicals

All manipulations were carried out under purified argon using the standard Schlenk technique. Reagent grade solvents were appropriately distilled and dried before use. RuCl_3_.xH_2_O, NH_4_PF_6_, 1,4-bis(diphenylphosphino)butane, and 2,2′-bipyridine were purchased from Aldrich and used as received. The precursor *cis*-[RuCl_2_(dppb)(bipy)] was prepared as previously described [17]. The amino acids glycine, L-alanine, L-methionine, L-leucine, and L-aspartic acid were purchased from Stream and used without purification.

### Preparation of complexes

Typically, the complexes were synthesized by reacting the precursor *cis*-[RuCl_2_(dppb)(bipy)] (0.09 g, 0.1 mmol), dissolved in 25 mL methanol, with an excess of the amino acid (0.17 mmol). If the amino acid was not soluble in methanol, it was first dissolved in a minimum volume of hot water and slowly dripped into the methanol solution. The solution was refluxed and stirred for 3 h, and NH_4_PF_6_ (0.025 g, 0.16 mmol) was added. After 1 h, the solvent was removed, dichloromethane was added to dissolve the complex, and the mixture was filtered. The volume of the solution was reduced to approximately 2 mL, and ether was added; an orange solid was precipitated, isolated by filtration, washed well with H_2_O and diethyl ether, and dried under a vacuum. The yields of these syntheses were approximately 85–90%. Partial elemental analyses (Table 1) were carried out on the Department of Chemistry of the Federal University of São Carlos – UFSCar, in an instrument of CHNS staff EA 1108 of the FISONS. ^31^P{^1^H} NMR were recorded on a Bruker DRX 400 MHz. CD3OD was used as solvent. Thin layer chromatography (TLC) was performed on 0.25 mm silica gel pre-coated plastic sheets (40/80 mm) (Polygram SIL G/UV254, Macherey & Nagel, Düren, Germany) using toluene/methanol (9/1) as eluent. Details of the syntheses and characterization of the complexes were publication [16].

**Table 1:** Microanalyses data for the [Ru(AA)(dppb)(bipy)]^n+^ complexes.

Complexes	C%	N%	H%
[Ru(met)(bipy)(dppb)]PF_6_	52.87(52.55)	4.30(4.50)	4.75(4.43)
[Ru(gly)(bipy)(dppb)]PF_6_	53.16(53.26)	4.65(4.70)	4.57(4.50)
[Ru(leu)(bipy)(dppb)]PF_6_	55.12(54.92)	4.38(4.48)	5.05(5.20)
[Ru(aspac)(bipy)(dppb)](PF_6_)_2_	45.58 (45.60)	3.92 (3.85)	3.80 (4.00)
[Ru(ala)(bipy)(dppb)]PF_6_	53.71(53.61)	4.58(4.50)	4.62(4.82)

*calculated (found).

### Cell Culture

The murine sarcoma-180 tumor cells (S180) (ATCC# TIB-66) and normal murine fibroblast cells (L-929) (ATCC# CCL-1) were cultured in suspension in RPMI 1640 and DMEM media (Sigma Chemical Co., MO), respectively, supplemented with 10% fetal calf serum, 100 µg/mL penicillin, and 100 µg/mL streptomycin. The cultures were incubated in a humidified incubator (Thermo Scientific) at 37°C with 5% CO_2_.

### Cell viability assay (MTT assay)

The cytotoxic effects of the amino acid ruthenium complexes were evaluated using an MTT assay with S180 tumor cells and L929 normal cells as described previously [18]. Briefly, 1.0×10^5^ S180 cells and 2.0×10^4^ L929 cells were plated in 96-well tissue culture plates and treated with different concentrations of amino acid ruthenium complexes (0.2–200 µM) for 48 h. After treatment, 10 µL of MTT (5 mg mL^−1^) was added to each well, and the plates were incubated at 37°C for an additional 3 h. The purple formazan crystals were dissolved in 50 µL of SDS, and the absorbance was determined at 545 nm using a Stat Fax 2100 microplate reader (Awareness Technology, Palm City, FL, USA). The cell viability was calculated as follows: viability (%)  =  (absorbance of the treated wells)/(absorbance of the control wells) ×100. The IC_50_ (complex concentration (µM) that results in a 50% reduction in cellular viability) was obtained from dose-response curves using GraphPad Prism 4.02 for Windows (GraphPad Software, San Diego, CA, USA).

The IC_50_ values obtained from the MTT assay were used as a reference for the selection of the most promising amino acid ruthenium complex for further testing.

### Cell Cycle analysis

The S180 cells were treated with complex 2 (40 µM and 60 µM) for 24 h and 48 h. Briefly, 5.0×10^5^ cells were harvested by centrifugation, washed with phosphate-buffered saline (PBS), fixed with 70% (v/v) cold ethanol and stored overnight at −20°C. The fixed cells were washed with PBS and incubated with propidium iodide (PI; Sigma-Aldrich) containing 0.05% RNase. The samples were incubated at 4°C in the dark and analyzed by flow cytometry (FACS Calibur, BD Biosciences). The percentage of cells in the G0/G1, S, G2/M and sub-G1 phases was analyzed using ModFit software.

### Annexin V-FITC/PI double staining and analysis by flow cytometry

The cell death of S180 tumor cells was examined using the Annexin V-FITC Apoptosis Detection Kit (BD Biosciences) according to the manufacturer's instructions. S180 cells were treated with complex 2 (40 µM and 60 µM) for 24 h and 48 h. Briefly, 5.0×10^5^ cells were harvested and washed with PBS. The cells were re-suspended in 400 µL of binding buffer. Next, 5 µL of Annexin V-FITC and 1 µL of PI were added. Flow cytometric analysis was performed immediately after supravital staining. Data acquisition and analysis were performed on a flow cytometer (FACSCalibur, BD Biosciences) using Cell Quest software. The criteria for positivity in cells in early stages of apoptosis were Annexin V-positive and PI-negative, whereas the criteria for cells in the late stages of apoptosis were both Annexin V-positive and PI-positive.

### Analysis of mitochondrial membrane potential

The effect of complex 2 on the mitochondrial membrane potential (*ΔΨ*m) was measured using JC-1 dye (BD Biosciences) by flow cytometry according to the manufacturer's instructions. Briefly, 3.0×10^5^ S180 cells were treated with complex 2 (40 µM and 60 µM) for 24 h. After incubation, the cells were harvested and washed with PBS. The cells were then incubated with JC-1 dye (BD Biosciences) for 15 min at 37°C in the dark. The stained cells were washed, resuspended in assay buffer, and immediately analyzed by flow cytometry. The data were analyzed using the Cell Quest software.

### Measurement of caspase activity

Direct measurement of the activity of caspases 3, 8, and 9 were performed using the ApoTarget Caspase 3, 8, and 9 Protease Assay (Invitrogen) according to the manufacturer's recommendations. After treatment with complex 2 (40 µM and 60 µM) for 24 h, the cells were lysed with chilled cell lysis buffer. The protein concentration was then measured using the BSA Protein Assay Kit (BioRad). An aliquot of the protein extract (75 µg) was mixed with 50 µL of 2X reaction buffer supplemented with 10 mM DTT and the substrates of DEVD-pNA (caspase-3), IETD-pNA (caspase-8), or LEHD-pNA (caspase-9). The mixtures were then incubated for 2 h at 37°C. Subsequently, the formation of p-nitroanilide in the samples was measured using an ELISA microplate reader at 405 nm. The increases in the activities of caspase-3, caspase-8, and caspase-9 were determined by comparing the results with the control.

### Total RNA extraction and cDNA synthesis

The S180 cells were treated with 40 µM complex 2 for 6 h. Total RNA was extracted with Trizol reagent (Sigma-Aldrich, USA) following the manufacturer's protocol. Total RNA (2.0 µg) was used to produce complementary DNA (cDNA) with random primers (Applied Biosystems, USA) in a 20 µL reaction mixture according to the manufacturer's protocol.

### Real-time quantitative PCR

Real-time PCR was carried out using a Line Gene K (Bioer Technology) instrument. Real-time PCR reactions were carried out in a 20 µL reaction mixture, including 2 µL of cDNA, 10 µL of SYBR Green PCR Master Mix (LGC Biotechnology, UK), and 2.0 µL of 400 nM forward and reverse primers. The PCR program was initiated at 95°C for 15 min followed by 40 cycles of 95°C for 15 s, 55°C for 15 s, and 72°C for 30 s. The data were analyzed according to the comparative Ct method and were normalized to the β-actin reference gene expression in each sample. The primer sequences are shown in Table 2.

**Table 2:** Primer sequences used for the real time RT-qPCR assay.

Gene	Primer sequences
*β-actin*	F5′CACACCCGCCACCAGTTC3′ R5′ATTCCCACCATCACACCCTG3′(161 bp)
*Bax*	F5′GCTACAGGGTTTCATCCAGG3′ R5′GGAGACACTCGCTCAGCTTC3′(113 bp)
*Caspase 3*	F5′GGAGCTTGGAACGCTAAGAA3′R5′GTCCACTGACTTGCTCCCAT3′ (112 bp)
*Caspase 8*	F5′AGGTACTCGGCCACAGGTTA3′ R5′TGGGATGTAGTCCAAGCACA3′(137 bp)
*Caspase 9*	F5′TAGCTGGAACACTGGGCATTGAGT3′R5′AACATACCCATCGGTGCATTTGGC3′(146 pb)
*P53*	F5′TGGAAGACTCCAGTGGGAAC-3′ R5′TCTTCTGTACGGCGGTCTCT-3′(87 pb)

### Western blot analysis

The S180 cells were treated with 40 µM and 60 µM complex 2 for 24 h. Untreated cells maintained in media only were used as the control. After treatment, the cells were washed with PBS and lysed with lysis buffer (Invitrogen). The samples were stored at −20°C until further analysis. The protein concentrations of the resulting lysates were determined using the Bradford assay [19]. A total of 70 µg of protein extract was mixed with Laemmli loading buffer and subjected to 12% SDS-PAGE at 100 V for 2 h, followed by electroblotting to nitrocellulose membranes (0.45 µm) (Bio-Rad Laboratories, Inc.) at 400 mA for 1.5 h. The membranes were blocked with 3% non-fat dry milk in Tris-buffered saline with Tween (TBS-T) for 1 h and subsequently incubated overnight at 4°C with 1% non-fat dry milk in TBS-T containing a 1∶500 dilution of primary antibodies specific to Bak (sc-832, Santa Cruz Biotechnology Inc.) and β-actin (sc-1615, Santa Cruz Biotechnology Inc.). The membranes were washed in TBS-T, incubated with an HRP-conjugated IgG secondary antibody (Santa Cruz Biotechnology Inc., 1∶1000 dilution) for 1 h, and washed with TBS-T. The immunoreactive bands were detected using an ECL western blotting substrate (Super Signal West Pico Substrate, Thermo Scientific), and the ECL signals were captured using a CCD camera (ImageQuant LAS 4000 mini; GE Healthcare). The optical density of the target protein bands was measured using IMAGE J software downloaded from the NIH website (http://rsb.info.nih.gov.ij/) to compare the protein levels. Bak expression was normalized to the β-actin levels.

### Atomic force microscopy (AFM)

For the atomic force microscopy (TMAFM) experiment, the pBR322 plasmid was heated at 60°C for 10 minutes to obtain the OC form. The concentration of the stock solution was 1 mg/mL in HEPES buffer (4 mM HEPES (pH 7.4) and 2 mM MgCl_2_. Each sample contained 1 µL of pBR322 plasmid at a concentration of 0.25 µg/µL in a final volume of 40 µL. The amount of complex 2 added is expressed as *r_i_*. AFM samples were prepared by casting a 3 µL drop of test solution onto freshly cleaved muscovite green mica disks as the support. The drop was allowed to stand undisturbed for 3 minutes to favor the adsorbate-substrate interaction. Each DNA-laden disk was rinsed with Milli-Q water and was blown dry with clean compressed argon gas directed normal to the disk surface. The samples were stored over silica prior to AFM imaging. All AFM observations were made with a Nanoscope III Multimode AFM (Digital Instruments, Santa Barbara, CA). Nanocrystalline Si cantilevers (125 nm in length) with an average spring constant of 50 N/m terminating in conical-shaped Si probe tips (10 nm apical radius and cone angle of 35°) were utilized. High-resolution topographic AFM images of 2×2 µm areas of different specimens were obtained in air at room temperature (relative humidity <40%). The AFM was operated in intermittent contact mode at a rate of 1–3 Hz.

### Statistical analysis

Statistical analysis of the results was performed using an unpaired t test or one-way ANOVA followed by Dunnett's post hoc test or the Bonferroni post hoc test for multiple comparisons with a control. All statistical analyses were performed using the statistical software GraphPad Prism (version 4). A probability of 0.05 or less was deemed statistically significant. The following notation is used throughout the manuscript: *, p<0.05 and **, p<0.01 relative to the control.

## Results

### Synthesis of the Ru(II) amino acid complexes

All the synthesized complexes are of orange color, and their elemental analyses data suggest the formation of [Ru(AA)(dppb)(bipy)]PF_6_ species for the amino acids glycine, L-alanine, L-methionine and L-leucine and [Ru(AA)(dppb)(bipy)](PF_6_)_2_ for the L-aspartic acid. The complexes were characterized by elemental analyses (Table 1), and their purity, which were of >97% were determined by ^31^P{^1^H} NMR, and by TLC analysis [16].

### S180 cell viability

The amino acid ruthenium complexes were tested *in vitro* for their cytotoxic effects on the S180 tumor cell line and the L929 normal cell line using the MTT assay. As shown in Table 3, the five complexes inhibited the growth of the S180 tumor cell line, with IC_50_ values ranging from 22.53 µM to 50.18 µM. The complexes containing methionine and glycine were more active against S180 cells, with IC_50_ values of 22.53 µM and 31.15 µM, respectively. The complex containing glycine showed lower toxicity against the L929 cells (51.65 µM) compared with the complex containing methionine (27.39 µM). As shown in Table 3, under the same conditions, cisplatin showed cytoxicity at concentrations as low as 29.05 µM, while its IC_50_ against S180 cells was 64.83 µM.

**Table 3:** Viability of the S180 tumor cell line and the L929 normal cell line in the presence of ruthenium amino acid complexes and cisplatin.

IC_50_ (µM)		
	L929	S180
Complex 1	27.39±3.32	22.53±3.40
Complex 2	51.65±1.22	31.15±4.19
Complex 3	47.15±5.94	38.19±7.00
Complex 4	106.72±8.98	42.22±3.98
Complex 5	97.75±8.99	50.18±1.03
Cisplatin	29.05±1.88	64.83±0.17

### Induction of G0/G1 arrest in S180 cells

**Figure 1:**
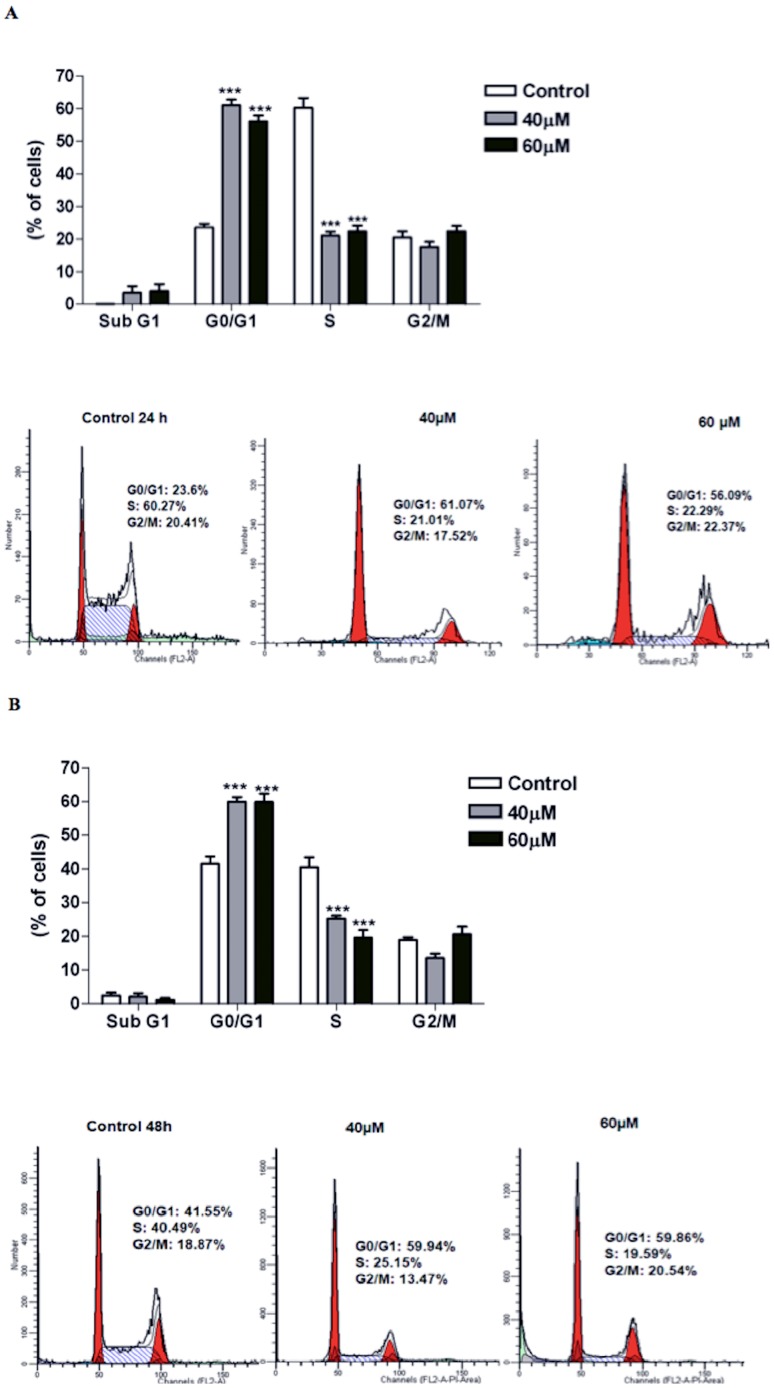
Effects of complex 2 on the cell cycle distribution of S180 cells after 24 h (A) and 48 h (B) of exposure. The data are the means ± SD of three experiments. Significant differences from the untreated control are indicated by ***p<0.001.

The cell cycle distribution of the S180 cells treated with complex 2 (40 µM and 60 µM) was examined by flow cytometry and revealed G0/G1 phase arrest (Fig. 1). As shown by the histograms of cell cycle distributions (Fig. 1 A, B), treatment with the complex caused a statistically significant increase in the proportion of cells in the G0/G1 phase, correlating with a decreased number of cells in the S phase (p<0.001). The fraction of G0/G1 cells increased from 23.6% (24 h) and 41.55% (48 h) in the control to 61.07% (24 h) and 59.94% (48 h) after treatment with 40 µM complex 2 and to 56.09% (24 h) and 59.86% (48 h) in cells treated with 60 µM complex 2.

### Induction of apoptosis in S180 cells

**Figure 2:**
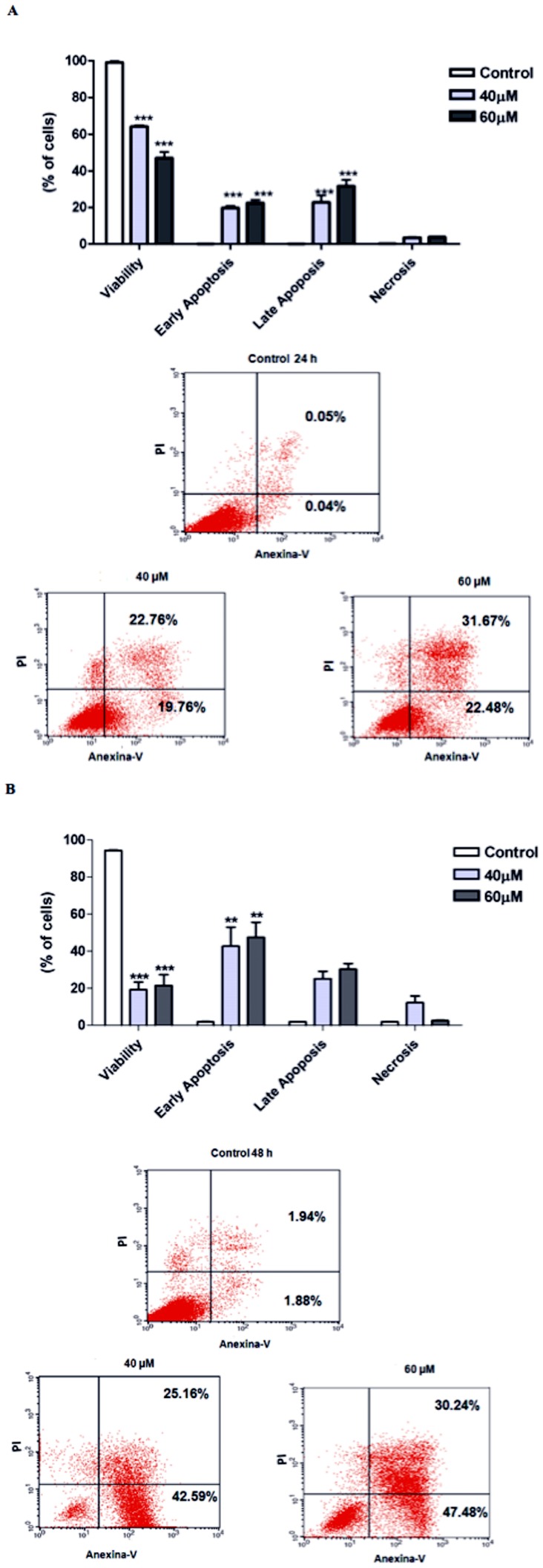
Effect of complex 2 on the mechanism of S180 cell death (early and late apoptosis/necrosis) evaluated by flow cytometry after 24 h (A) and 48 h (B) of incubation. The data are the means ± SD of three experiments. Significant differences from the untreated control are indicated by **p<0.01 and ***p<0.001.

To evaluate the mechanism of S180 cell death, the FITC-Annexin V/PI assay was used. A total of 19.76% and 22.48% of the cells treated with 40 µM and 60 µM complex 2, respectively, were in early apoptosis (Annexin V+ and PI-) at 24 h, and 42.59% and 47.48% of the cells, respectively, were in early apoptosis at 48 h (p<0.001 compared to the control). Additionally, a total of 22.76% and 31.67% of the cells treated with 40 µM and 60 µM complex 2, respectively, were in late apoptosis (Annexin V+ and PI+) at 24 h, while 25.16% and 30.24% of the cells, respectively, were in late apoptosis at 48 h. Figure 2A (24 h) and B (48 h). (p<0.001 compared to the control).

### Disruption of the mitochondrial membrane potential

**Figure 3:**
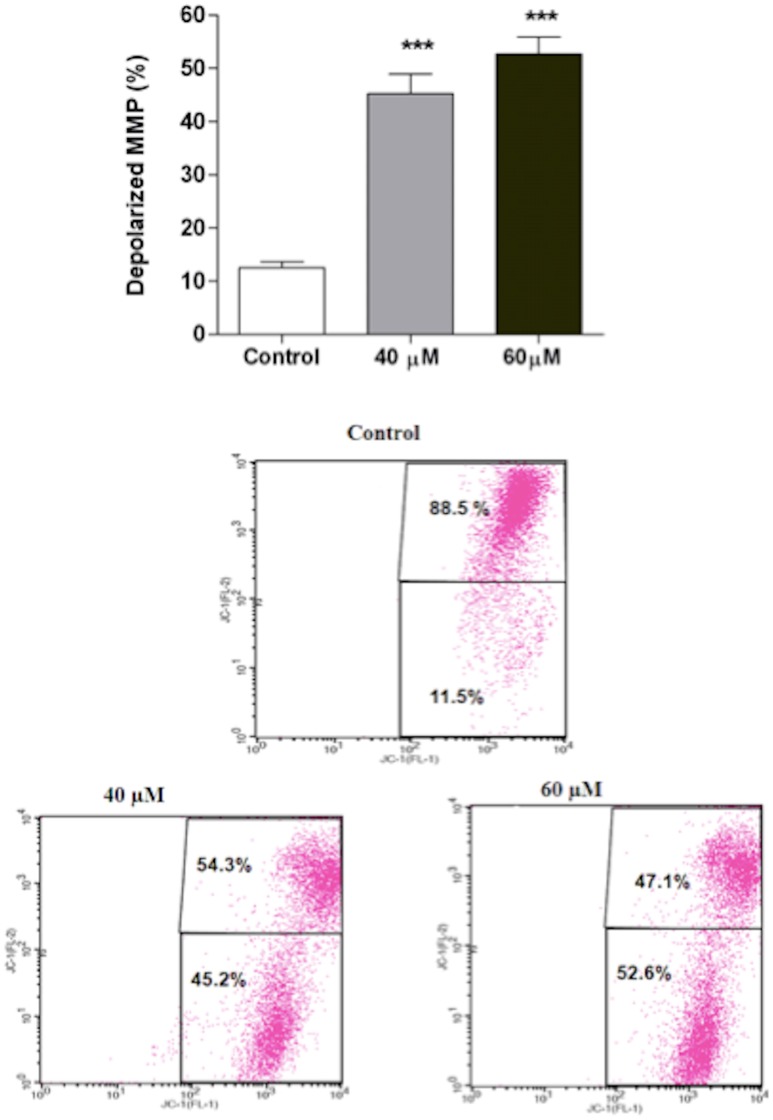
Effect of complex 2 on the mitochondrial membrane potential. The percentages of cells with depolarized mitochondrial membrane potential mitochondrial membrane potentials of cells treated with 40 µM and 60 µM complex 2 as well as control cells were assessed by flow cytometry after staining with JC-1. The data are expressed as the means ± SD of two individual experiments; ***p<0.001 compared with the control.

To evaluate the possible effect of the complex 2 on the mitochondrial membrane potential (*ΔΨ*m), JC-1 staining was performed. We observed that after treatment with complex 2 for 24 h, significant loss of *ΔΨ*m was induced in the S180 cells. The percentage of cells with depolarized mitochondria increased from 11.5% (control) to 45.2% (40 µM) and 52.6% (60 µM). Fig. 3 shows typical FL-1 (green fluorescence) and FL-2 (red fluorescence) dot plots for JC-1-stained S180 cells treated with complex 2 (40 and 60 µM) and untreated control cells. Representative dot plots of the effect of complex 2 on the mitochondrial membrane potential are shown in Fig. 3. It was found that JC-1 aggregates accumulated in the control cells and displayed a high red signal (top right quadrant). Treatment of the cells with complex 2 for 24 h resulted in a shift to higher green fluorescence intensity, with a concomitant decrease in the percentage of cells showing red fluorescence to 54.3% (40 µM) and 47.1% (60 µM) of cells (upper-right quadrant).

### Induction of S180 cell apoptosis through a caspase-dependent pathway

**Figure 4:**
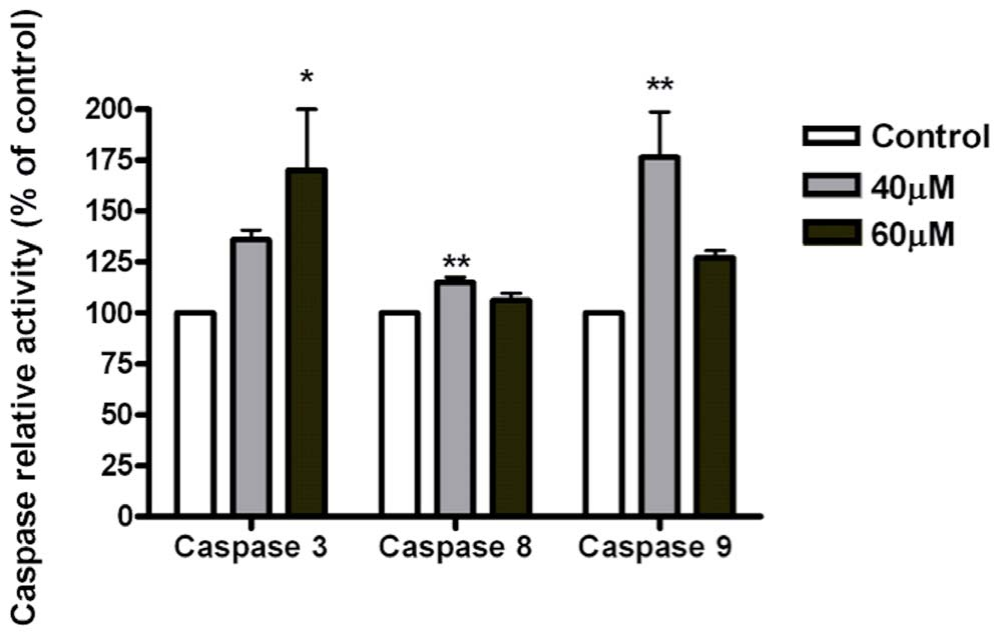
Effect of complex 2 on the activity of caspase -3, -8 and -9 in S180 cells. S180 cells were treated with the complex at the indicated concentrations for 24 h. The data are expressed as the means ± SD of two individual experiments; *p<0.05 and **p<0.01 compared with the control.

After treatment with the complex 2 for 24 h, the activity of caspases 3, 8, and 9 was increased in the S180 cells compared to the control. Treatment of cells with complex 2 (60 µM, 24 h) significantly (p<0.05) increased caspase-3 activity. The activity of caspase-3, increased from 100% in the control to 136.05% and 170.24% in cells treated with 40 µM and 60 µM, respectively (Fig. 4). The activities of caspase -8 and caspase-9, after treatment with complex 2 (40 µM, 24 h), increased from 100% in the control to 127.22% and 176.43%, respectively.

### Induction of the expression of apoptosis-related genes

**Figure 5:**
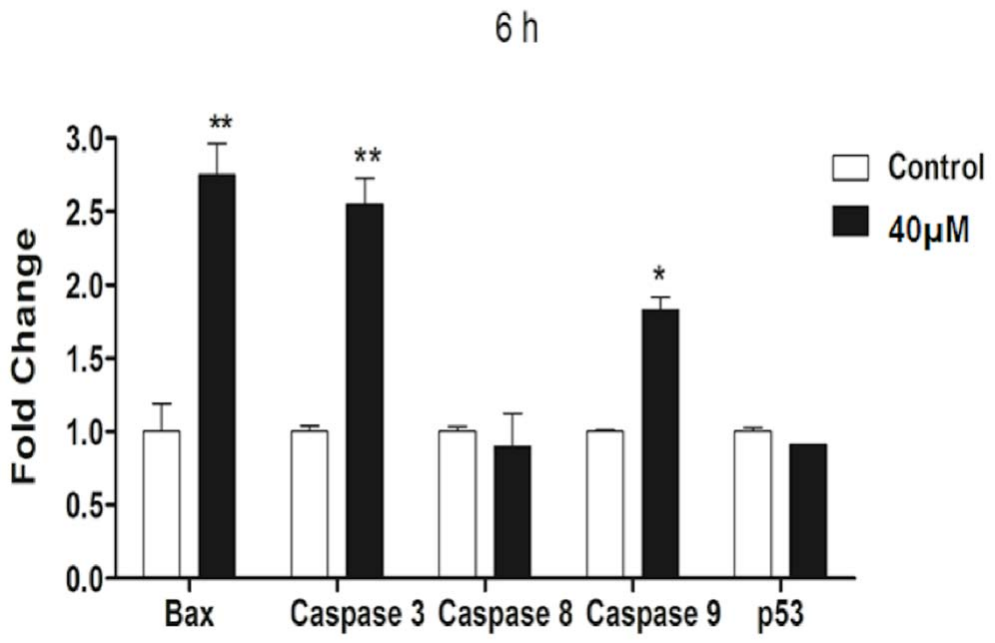
Relative expression of *bax*, *caspase 3*, *caspase 8*, *caspase 9*, and *p53* in S180 cells treated with 40 µM of complex 2 for 6 h as determined by real-time RT-PCR analysis. The fold change is expressed relative to the respective untreated control using *β-actin* as a reference gene. The data are expressed as the means ± SD; * p<0.05, **p<0.01, and ***p<0.001 compared with the control.

he effect of complex 2 on the expression of apoptosis-related genes was evaluated by real time RT-PCR analysis. Fig. 5 shows the gene expression levels of bax, caspase 3, caspase -8, caspase 9, and p53 after incubation with 40 µM complex 2 for 6 h. Treatment of S180 cells with the complex significantly increased bax, caspase 3, and caspase 9 gene expression. The expression level of the bax gene increased 2.75-fold compared to its level in untreated control cells. The expression levels of caspase 3 and caspase 9 increased significantly by 2.55- and 1.83-fold, respectively, compared to the control (p<0.05). The expression levels of caspase 8 and p53 remained unchanged after treatment with the complex 2 compared with the control (p>0.05).

### Expression of the apoptosis-related Bak protein in S180 cells

**Figure 6:**
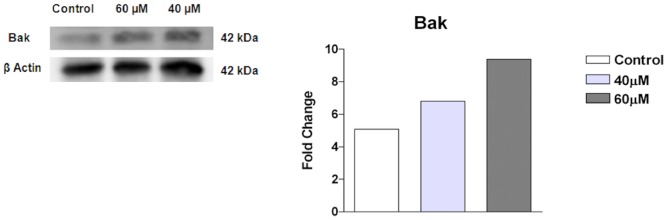
Western blot analysis of Bak protein expression in S180 cells after incubation with 40 and 60 µM complex 2 for 24 h. β-actin was used as a control.

The expression of the Bak protein was detected by western blot analysis. When S180 cells were treated with 40 µM and 60 µM complex 2 for 24 h, a concentration-dependent increase in Bak expression was observed. As shown in Fig. 6, the expression level of the Bak protein increased from 5.08- (control) to 6.81- and 9.40-fold after incubation with 40 µM and 60 µM complex 2, respectively.

### Atomic force microscopy

**Figure 7:**
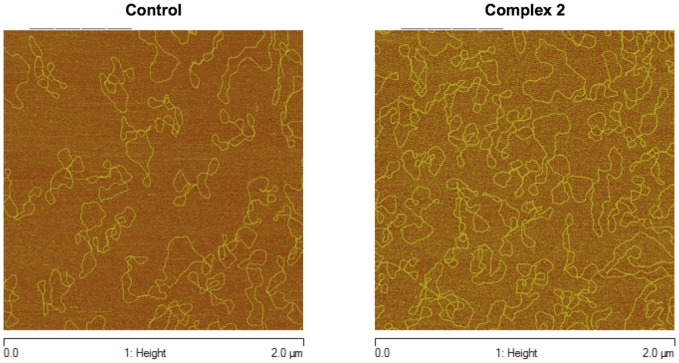
AFM image of free pBR322 plasmid DNA (A). AFM image of the pBR322 plasmid incubated with the complex 2 for 24 h at 37°C (B).

AFM images of free pBR322 plasmid DNA and pBR322 plasmid DNA incubated with complex 2 are shown in Fig. 7A and B, respectively. The images show that the complex 2 did not covalently bind to DNA bases or intercalate between bases; however, there was a weak interaction that caused increased coiling of the DNA compared to the free pBR322 plasmid DNA.

## Discussion

In this study, we investigated the cytotoxic activity of ruthenium/amino acid complexes against the S180 tumor cell line and the L929 normal cell line using the MTT assay. The IC_50_ values of the five amino acid complexes and cisplatin (positive control) are shown in Table 2. Based on the IC_50_ values, the *in vitro* cytotoxic activities of the complexes were ordered as follows: complex 1> complex 2> complex 3> complex 4> complex 5. Additionally, all the complexes were more cytotoxic than cisplatin. The complexes containing aspartic acid, alanine, glycine, and leucine were less cytotoxic toward the L929 murine fibroblast cells, with IC_50_ values ranging from 106.72 µM to 47.15 µM; these values were significantly higher than the IC_50_ for cisplatin (29.05 µM) (Table 2). These results suggest that the complexes studied in this work are generally more selective for cancer cells compared with fibroblast cells. In this study, the complex 2 was shown to be the most promising compound in the series, showing the highest activity against the S180 tumor cells and lower cytotoxicity against the L929 normal cells.

The inhibition of cell viability by cytotoxic drugs can result from cycle cell arrest, induction of apoptosis, or a combination of these two mechanisms [13], [19]. Flow cytometric analysis was performed to determine the possible mechanisms of cell growth inhibition by complex 2 in S180 cells. As shown in the cell cycle analysis, exposure of the S180 cells to complex 2 led to a significant increase in the percentage of cells in the G0/G1 phase, which was accompanied by a corresponding reduction in the percentage of cells in the S and G2/M phases. During chemotherapy, the accumulation of cells in the G0/G1 phase is often the result of cell cycle checkpoint activation as a consequence of DNA damage. Previous studies have showed that some ruthenium compounds induce the arrest of cells in the G0/G1 phase through p53 activation and an increase in the protein levels of p21, an inhibitor of the cell cycle that blocks CDK activity [20]–[22]. Molecularly, this process is often mediated by the activation of p53, which results in cell cycle arrest predominantly in the G1 phase and induction of apoptosis. To confirm whether the complex 2 caused p53 activation, we analyzed the expression of the p53 gene by real time RT-PCR. Our results showed that the treatment of the S180 cells with the complex 2 did not increase the expression level of p53 after 6 h of incubation, indicating that the complex 2 can act on these checkpoints through p53-independent mechanisms, preventing cell division and subsequently initiating apoptosis.

To determine whether the growth inhibitory activity of the complex 2 was related to the induction of apoptosis, we carried out a cell death assay using Annexin V staining. This assay showed that treatment with complex induced apoptosis in S180 cells as shown by a significant increase in Annexin V-positive cells. Consistent with our results, previous studies have demonstrated that the *cis*-[RuCl_2_(NH_3_)_4_]Cl ruthenium compound also increased the numbers of Annexin V-positive S180 cells [17].

The caspases, a family of cysteine proteases, have been shown to act as central executioners of the apoptotic pathway [23]. To study the involvement of caspases in the signaling pathways involved in complex 2-induced apoptosis, the activities of the effector caspase-3 and two initiator caspases, caspase-8 and caspase-9, were investigated using a colorimetric assay, and the level of mRNA expression of these caspases was investigated by real time RT-PCR analysis. In the caspase activity assay, treatment with the complex 2 resulted in a significant increase in the activity of caspase-3 and caspase-9 in S180 cells, indicating the involvement of caspase-dependent mechanisms in complex 2-induced apoptosis. The mRNA expression analysis revealed that the complex caused a rapid and significant increase in the expression of caspase-3 and caspase-9, which corroborated the caspase activity results.

Previous reports have shown that Ru(II) and Ru(III) complexes trigger intrinsic and extrinsic apoptosis pathways [13], [23], [24], and it has recently been shown that ruthenium complexes containing 2,6-bis(benzimidazolyl) pyridine induce apoptosis through caspase -3-, caspase -8-, and caspase -9-dependent pathways in A375 human melanoma cancer cells [22].

Mitochondrial dysfunction can induce the release of apoptogenic factors, such as cytochrome c, from the mitochondrial inner space into the cytosol, triggering apoptotic pathways. The loss of mitochondrial membrane potential, an early event in apoptosis, represents mitochondrial dysfunction, which is an irreversible checkpoint in apoptosis. Loss of ΔΨm is associated with the activation of caspases and the initiation of apoptotic cascades [25], [26]. Thus, in this study, to determine the role of the mitochondria in complex 2-induced apoptosis, we examined the loss of mitochondrial membrane potential using the JC-1 assay. As indicated in Fig. 3, treatment of S180 cells with complex induced a significant loss of ΔΨm, suggesting that mitochondria play an important role in complex 2-induced apoptosis. Ruthenium complexes exhibit cytotoxic activity against various types of tumor cell lines, affecting mitochondria and inducing caspase activation [27]. Taken together, our data indicate that the loss of mitochondrial potential and the activation of caspases are crucial for complex 2-induced apoptosis in S180 cells.

Bcl-2 family members are important regulators of mitochondria-mediated apoptosis. These proteins can be targeted to the mitochondria and regulate mitochondrial membrane permeabilization during apoptotic events. Among the Bcl-2 family members, Bcl-2 and Bcl-xL are anti-apoptotic members, whereas Bax and Bak are pro-apoptotic members. Bax and Bak serve as a necessary gateway for the release of apoptotic proteins such as cytochrome c and Smac [27], [28]. Interestingly, in the present study, our results showed that the levels of Bak (protein) and Bax (mRNA) were increased after treatment with complex, further confirming the that the intrinsic mitochondrial pathway triggers complex 2-induced S180 cell apoptosis. Based on these results, the increases in the levels of Bax and Bak may trigger the depolarization of the mitochondrial membrane potential, as evidenced by the JC-1 assay, and consequently induce apoptosis. These results are consistent with previous reports, which have shown that ruthenium complexes increase Bax levels, with consequent alterations in the mitochondrial membrane potential [21], [29].

In a previous study [30], we reported that treatment with the [Ru(pic)(dppb(bipy)]PF_6_ (pic  = 2-pyridinecarboxylic acid anion) complex resulted in the strong aggregation of plasmid DNA, as shown by AFM images. No nuclease activity was observed, and the modifications observed confirmed that the complex did not destroy the structure of the pBR322 plasmid DNA but rather yielded more coiled and/or more associated plasmids. Thus, a similar AFM image was obtained for the complex 2 (Figure 7), which revealed that this complex does not allow either covalent binding to DNA bases or intercalation between the bases; however, there was a weak interaction that caused greater coiling of the DNA compared to the free pBR322 plasmid DNA. Thus, these AFM data suggest that the complex 2 can also signal apoptosis through DNA pathways.

## Conclusions

In summary, our results showed that amino acid ruthenium complexes are potent inhibitors of S180 tumor cell viability. The complex 2 inhibits cell growth through G0/G1 phase arrest and induction of apoptosis. Further investigation showed that the complex 2 caused a loss of mitochondrial membrane potential; induced the activation of caspases 3, 8, and 9; and caused changes in the expression levels of the pro-apoptotic Bcl-2 family, Bax (mRNA) and Bak (protein). However, the mRNA expression level of p53 remained unchanged.

**Figure 8:**
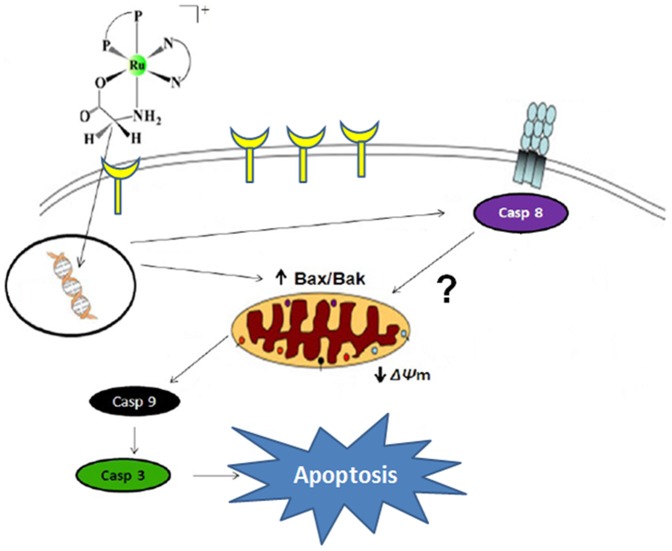
Proposed apoptotic signaling pathways triggered by complex 2 in S180 tumor cells.

In Figure 8, we propose a model for the apoptotic signaling pathway that is triggered by the complex 2 in S180 tumor cells.
